# The Influence of Milk Leakage, Udder Pressure and Further Risk Factors on the Development of New Intramammary Infections during the Dry Period of Dairy Cows

**DOI:** 10.3390/pathogens13050430

**Published:** 2024-05-20

**Authors:** Pauline Katthöfer, Yanchao Zhang, Nicole Wente, Franziska Preine, Julia Nitz, Volker Krömker

**Affiliations:** 1 Department of Microbiology, Faculty of Mechanical and Bioprocess Engineering, University of Applied Sciences and Arts, 30453 Hannover, Germany; pauline.katthoefer@hs-hannover.de (P.K.); yanchao.zhang@hs-hannover.de (Y.Z.); nicole.wente@hs-hannover.de (N.W.); franziska.preine@hs-hannover.de (F.P.); julia.nitz@hs-hannover.de (J.N.); 2 Department of Veterinary and Animal Sciences, University of Copenhagen, 1870 Frederiksberg C, Denmark

**Keywords:** cattle, mastitis, udder health, drying off, milk leakage, udder pressure, udder hygiene, risk factors

## Abstract

Prevention of new intramammary infection (NIMI) during the dry period (DP) is essential to prevent the development of mastitis in dairy cows. To investigate risk factors for NIMI, 212 cows, comprising a total of 848 udder quarters, were examined in this study. Quarter milk samples were taken on the day of drying off and 7 ± 3 days after calving. Cow- and quarter-level associated risk factors were assessed at the beginning of the DP and after calving. In total, 7.1% of the udder quarters developed an NIMI between the samplings. Non-*aureus* staphylococci (40.4%) and Gram-negative pathogens (22.8%) were most frequently the cause of NIMI. The observed milk leakage prevalence was 16.7%, with a peak 24 h after drying off. Simultaneously, the udder pressure peaked 24 h after drying off. A significant correlation between milk yield on the day before drying off and milk leakage could be proven. Cows with quarters leaking milk produced an average milk yield of 28.32 kg on the day before drying off. Generalised linear mixed models and odds ratios were calculated to determine the significant risk factors for NIMI during the DP and early lactation. Quarters leaking milk had 3.4 higher odds for NIMI between the samplings compared to quarters without milk leakage. Quarters from cows with dirty udders had 3.1 higher odds of developing an NIMI between the samplings compared to quarters from cows with clean udders. The results of this study demonstrate the importance of dry cow management before drying off and during the critical period of active involution of the udder tissue.

## 1. Introduction

The period between drying off and calving, the dry period (DP), is an important part of the reproductive cycle of dairy cows not only for the gestation but also considering udder health. On the one hand, the bovine mammary gland can recover from the previous lactation and the DP offers the opportunity to cure existing intramammary infections. Cure rates of up to 86% are reported [1,2]. On the other hand, the DP carries the risk of new intramammary infections (NIMIs), primarily with environmental pathogens [3,4]. In many cases, intramammary infections developed during the DP lead to clinical mastitis (CM) at the beginning of the following lactation [5]. Main economic losses caused by mastitis arise through treatment costs, discarded milk and a decline in milk yield [6,7]. Another negative impact of mastitis is the increased risk of culling and death of the affected cow regardless of the mastitis-causing pathogen [8,9]. To avoid NIMI and thus mastitis, it is important to know the risk factors that lead to NIMI to take advantage of the DP. Due to the genetic selection of high milk yield, advanced management and nutrition, the milk production of dairy cows rises constantly [10]. Stefanon et al. [11] have already drawn attention to the high milk yield of 25–30 kg/day on the day of drying off. Nevertheless, most of the dairy cows are still dried off abruptly and the dry cow management has not been adapted to the increased milk yield [12]. The European Union Regulation 2019/6 has also banned routine blanket dry cow treatment since 2022, which has led to changes in dry cow management [13]. The DP can be divided into three phases: the active involution, the steady state and, 15 to 20 days antepartum, the neo-lactogenesis [14]. The udder tissue is particularly vulnerable to NIMI during active involution and neo-lactogenesis. After dry-off treatment, the milking is commonly ceased abruptly [12]. However, the udder tissue continues to produce milk in the first two to three days after drying off. Milk accumulates in the udder and is an appropriate culture medium for bacterial growth. Additionally, the flushing effect of the usually regular milking is omitted [14]. Moreover, the accumulating milk causes the udder pressure to rise. Due to a high intramammary pressure, the milk secretion declines; however, previously a high udder pressure can lead to milk leakage [15,16,17]. The keratin plug, a physical barrier that is formed in the teat canal, physiologically prevents milk leakage, protects the mammary gland from pathogens entering and inhibits bacterial growth through its fatty acids [18,19]. Dingwell et al. [19] observed that cows yielding >21 kg milk at drying off had fewer closed teats after drying off because no keratin plug had formed. De Prado-Taranilla et al. [20] demonstrated that cows with milk leakage tended to be 1.5 times more susceptible to NIMI in contrast to cows without milk leakage. However, milk leakage is only one potential risk factor for the development of NIMI. Mastitis and NIMI are caused by the influence of multiple factors. Their development is affected by the exposure to pathogens, the environment and the individual animal [21]. Detected cow- and quarter-level associated risk factors are, for example, poor teat and udder hygiene, hyperkeratotic teat end condition, teat shape and a large variation in the body condition during the DP [4,22,23]. 

Nevertheless, knowledge about the influence of high milk yields on the day of drying off on udder pressure, milk leakage and pathogens leading to NIMI is limited. As presented, there is a high risk for the development of NIMI at the beginning of the DP and at the end of the DP and early lactation also has an important influence on udder health. The conducted study not only includes the time from drying off to calving, but also considers an extended dry period of 7 ± 3 days after calving (DP+ calving week). The study aimed to capture the association between milk yield, udder pressure, milk leakage prevalence and risk factors for NIMI during the DP+ calving week. Therefore, the study investigated risk factors for NIMI during these crucial periods [4]. 

## 2. Materials and Methods

### 2.1. Herd and Cow Selection

For the cross-sectional study, three conventional dairy farms were visited over a period of six months from August 2022 to January 2023. Farms were selected on the basis of their confirmation of participation and location close to Hannover, Germany. Two farms were located in Lower Saxony (Farm A and B) and one was located in North Rhine-Westphalia (Farm C), Germany. All three farms were participating in the local dairy herd improvement (DHI) programme. Farm A milked 528 dairy cows three times a day in a 2 × 16 side-by-side milking parlour with an average energy-corrected yearly milk production (ECM) of 12,717 kg. Farm B milked 311 cows in a 2 × 20 side by side milking parlour twice a day with an average yearly ECM of 9267 kg. Farm C milked 160 cows twice a day in a 2 × 10 herringbone milking parlour with an yearly ECM of 11,300 kg. The average somatic cell count (SCC) of the last DHI before the start of the trial amounted to 201,000, 214,000 and 158,000 cells/mL, respectively, for farms A, B and C. 

In total, 848 quarters from 212 Holstein Friesian cows were included in this study. All dairy cows included were dried off between August 2022 and November 2022, had four functional udder quarters and had not been treated with antimicrobials in the previous 30 days. 

All 212 dairy cows were abruptly dried off by applying a long-lasting intramammary antibiotic and an internal teat sealer. The drying off management on the farms was not changed for the trial. Farm B applied a long lasting intramammary antibiotic with the active substance cloxacillin-benzathin (Cloxacillin-Benzathin, CP-Pharma Handelsgesellschaft mbH, Burgdorf, Germany). Farm A and C treated the udder quarters with a combination of benethamine penicillin, penethamate hydriodide and framycetin sulphate (Benestermycin, Boehringer Ingelheim Pharma GmbH & Co. KG, Ingelheim, Germany). Finally, all teats were dipped with iodinated teat dip. After drying off, the cows were separated from the herd. On farm A and C, the dry cows were housed in a free stall barn; on farm B, dry cows were housed in deep-bedded straw yards.

General information about the cows like age, lactation number, duration of the current lactation and the duration of the DP were collected. Farm A and B had milk meters installed in their milking parlours to record the milk yield and the milk flow of each cow at each milking. Farm C also measured the milk yield of the respective cows one day before drying off but in a milk can. The milk yield (kg) from the day before drying off was documented for the trial.

### 2.2. Quarter Milk Samples

Between August 2022 and January 2023, the farms were visited by a veterinarian for quarter milk sampling. For each dairy cow included in this study, quarter milk samples were collected twice on the days of drying off and 7 ± 3 days after each calving. For the second sampling, the milk had to be free of colostrum characteristics. Colostrum was identified by its yellowish colour and thick, sticky consistency. A reliable mastitis diagnosis is thus possible from day 3 after calving [24]. Quarter milk samples were taken as close as possible to the 3 days postpartum. Milk samples were collected in accordance with the guidelines of the German Veterinary Medical Association [25]. Quarter milk sampling was performed on both sampling dates before normal milking. The udder quarters were dry cleaned to remove coarse dirt. Three milk streams were milked and discarded from each teat. Subsequently, the teat apex and teat orifice were cleaned with 70% ethanol. For the milk sampling, the veterinarian wore disposable gloves. To reduce contamination, the sampling tube was opened under the udder, held horizontally and closed again under the udder after filling. The collected milk samples were stored in a cooling box and transported to the laboratory (Department of Microbiology, Faculty of Mechanical and Bioprocess Engineering, Hannover University of Applied Sciences and Arts, Hannover, Germany) on the day of sampling for cytomicrobiological examinations. 

### 2.3. Laboratory Procedures

The cytomicrobiological examinations of the milk samples were performed at the Laboratory of Microbiology, Faculty of Mechanical and Bioprocess Engineering, Hannover University of Applied Sciences and Arts in accordance with the guidelines of the German Veterinary Medical Association [25]. The test tubes contained the preserving agent boric acid (Ly20) [26]. Milk samples were stored at 4 °C until analysis. Ten µL of each shaken milk sample was applied to one quadrant of an esculin blood agar plate (5% defibrinated sheep blood, Oxoid Deutschland GmbH, Wesel, Germany). The plates were incubated at a temperature of 37 °C. The examinations were implemented after 24 h and after 48 h of aerobic incubation. Gram staining was performed to differentiate between Gram-positive and Gram-negative bacteria. A preliminary assessment was made by colonies morphology, catalase reactivity (3% H_2_0_2_; Merck KGaA, Darmstadt, Germany), esculin hydrolysis and hemolysis type. Afterwards, biochemical tests were performed to identify the microorganism. To verify bacterial genus and species, matrix-assisted laser desorption ionisation-time of flight (MALDI-TOF) was performed thereafter. All differentiated pathogens except for yeasts, *Prototheca*, *Pseudomonas* spp. and *Bacillus* spp. were analysed by MALDI-TOF (Microflex LT/SH smart, Bruker Daltonik, Bremen, Germany). A representative colony was used for MALDI-TOF analysis as described by Randall et al. [27]. Gram-positive and catalase-positive cocci were categorised as staphylococci. Beta-hemolysis and the clumping factor-test (Staph Plus Kit, DiaMondiaL, Virotech Diagnostics GmbH, Vienna, Austria) were used to differentiate between non-*aureus* staphylococci (NaS) and *Staphylococcus aureus*. *Staphylococcus aureus* showed beta-hemolysis and was clumping factor-positive. All clumping factor-negative colonies were determined to be NaS.

Gram-positive and catalase-negative cocci were categorised as streptococci or enterococci. Esculin non-hydrolysing, beta-hemolytic streptococci were classified by Lancefield serotyping (Dia-MondiaL Streptococcal Extraction Kit Sekisui, Virotech Diagnostics GmbH, Dietzenbach, Germany). Streptococci from group C were referred to as *Streptococcus dysgalactiae*, whereas streptococci from group B were referred to as *Streptococcus agalactiae*. Esculin hydrolysing streptococci were plated on modified Rambach agar to check for beta-d-galactosidase activity. Beta-d-galactosidase-positive colonies were identified as *Streptococcus uberis*, beta-d-galactosidase-negative colonies as *Enterococcus* spp. Gram-positive, beta-hemolytic, and catalase-negative, V- or Y- shaped rods were identified as *Trueperella pyogenes*. Gram-positive, catalase-positive, irregular rods were identified as coryneform bacteria. Yeasts, moulds and *Prototheca* were differentiated microscopically by their cell morphology after 48 h incubation of the esculin blood agar plates. *Bacillus* spp. were identified by their colony morphology. *Pseudomonas* spp., gram-negative, catalase-positive, were cytochrome oxidase C-positive and showed oxidative glucose degradation. Gram-negative, catalase-negative, cytochrome oxidase C -negative colonies were determined as *Escherichia coli*, *Klebsiella* spp. and other coliforms. Differentiation was based on cytochrome oxidase C-negativity and fermentative glucose degradation. Pure cultures were plated again on Chromocult coliform agar (Merck KGaA, Darmstadt, Germany) and incubated for 24 h at 37 °C. *Escherichia coli* grows blue colonies. Pink-growing colonies were either *Klebsiella* spp. or other coliforms. Enterobacteriaceae grows white to colourless colonies. To confirm *Klebsiella* spp., the oxidative/fermentation glucose test (OF glucose test) was performed. *Klebsiella* spp. showed no motility. 

Milk samples were classified as microbiologically positive if ≥5 colonies from the same environmentally associated mastitis-causing microorganism were growing on the plates. If two pathogens were cultured, a mixed infection was present. For cow-associated microorganisms like *Staphylococcus aureus*, *Streptococcus agalactiae* and *Streptococcus dysgalactiae*, one colony was sufficient to confirm an intramammary infection. A milk sample was considered contaminated if >2 different pathogens were growing on the plate.

The SomaScope TM Smart (Delta Instruments B.V., Drachten, The Netherlands) was used to measure the somatic cell count of the milk samples by flow cytometry. 

### 2.4. Data Collection

Potential risk factors for NIMI during the DP and early lactation were collected by literature research. Table 1 gives an overview of cow-associated risk factors and Table 2 illustrates quarter-associated risk factors for NIMI. 

Assessment of the risk factors for NIMI during the DP+ calving week was performed on the day of drying off, during the DP and 7 ± 3 days after calving.

Udder hygiene was scored using the scoring chart published by Schreiner and Ruegg [40]. Udders free of dirt were assigned to score 1. Score 2 describes slightly dirty udders with 2–10% dirt on the surface. If the surface was covered with 10–30% dirt, the udder was classified as score 3. The last score (4) describes an udder covered with caked-on dirt, where more than 30% of the surface was covered. Udder hygiene scoring was implemented five times by a veterinarian: on the day of drying off, 24 h, 48 h, 72 h after drying off and after calving. 

Teat cleanliness was scored before dry teat cleaning for quarter milk sampling, on the day of drying off and 7 ± 3 days after calving. Cleanliness was scored in the milking parlour by using the Teat Cleanliness Scorecard, describing the amount of manure, dirt and dip on the teat. The scores describe a clean teat free of manure dirt or dip (1), dip on the teat, but no manure or dirt (2), small amounts of dirt and manure (3) or large amounts of dirt and manure (4) [44]. 

Teat end condition was observed once for each teat on the day of drying off after milking. Classification of the teat end hyperkeratosis was made by using the classification system of Mein et al. [45]. Teat end conditions showed either no ring of hyperkeratosis (N), were encircled by a raised ring, with slight or no roughness (S), showed orifices surrounded by a raised rough ring and old keratin shreds (R) or showed a raised ring around the teat opening, rough and cracked (VR).

Furthermore, on the day of drying off the teats were measured. Teat length, circumference and height above the floor were measured while the cows were standing in the milking parlour, directly after milking and after the long-lasting intramammary antibiotic and the teat sealer had been applied. After drying off, the cows stood in the milking parlour for approximately 30 min on Farm A and Farm B. Farm C applied the dry-off treatment during the daily milking process.

The shape of the teat tip was classified on the day of drying off. The classification was based on the scheme of Grunert et al. [46], whereby the teats were divided into four groups: normal teat with rounded end (1), plate-shaped teat with flattened end on which two concentric annular rings form a disc (2), funnel-shaped teat with funnel around the orifice of the teat canal (3) and pointed teat with pointed end (4).

Udder pressure was measured using a modified handheld algometer. The Commander echo algometer from JTECH Medical Industries Inc. (Midvale, UT, USA) allows a non-invasive pressure measurement. The algometer consists of a measuring tip, a pressure sensor, a processing unit and a digital display. The threshold of the algometer was 0.2 kg/cm^2^;. The measuring point was marked with an animal marker to ensure the exact same measuring point for all measurements. The middle point of the two rear quarters was described as reflecting the udder pressure with the fewest deviations [47]. To gain accurate measured values, the method of Bertulat et al. [48] was followed. The cow had to stand still on a flat floor. The tip of the algometer was pressed at a right angle against the udder. Four measurements were performed for each rear quarter and the mean value was calculated by the algometer. If the coefficient of variation exceeded 10%, all eight measurements were repeated. By measuring the udder pressure after the last milking, a base value was documented. Every 24 h after drying off, at the same time as normal milking, the pressure was measured. A total of five measurements were taken. The first before the last milking, the second immediately after milking (base value) and again 24 h, 48 h and 72 h after drying off.

Milk leakage was observed three times at 24 h, 48 h and 72 h after drying off. Milk leakage observation was performed each day at the same time as the normal milking would have been performed. Milk leakage was scored as no milk leaking from the teats (0), the occurrence of milk under the udder (1), milk dripping from the teat (2) and milk flowing from the teat end (3). 

The body condition score was assessed twice for each cow during this study. The first assessment date was the day of drying off and the second one was 7 ± 3 days after calving, on the same day the quarter milk samples were taken. A scoring system was used as described by Edmonson et al. [49]. Eight body locations of the Holstein cows were considered when assigning them to the scoring system. A scale from one to five with 0.25 increments described an emaciated condition (1) up to an obese body condition (5). 

To assess the gait, before drying off and after calving, the 5-point lameness scoring system published by Sprecher et al. [50] was used. The posture and gait of the cows were scored. The lameness scoring starts with normal posture and gait (1), the cow stands and walks with a level-back posture, and the gait is normal. It continues with mildly lame (2), moderately lame (3), lame (4) and ends with severely lame (5) where the cow does not bear weight on one or more of her feet.

Udder oedema in this study was assessed once per cow, 7 ± 3 days after calving on the same day as the second milk samples were collected. By pressing the fingertip for 30 s into the oedema, the intercellular fluid is displaced and an indentation remains [51]. The udders were divided into udders with (1) or without (0) current oedema. 

In addition, the calving process was classified into calving alone without assistance (1), calving with assistance, either with less support (2) or with difficult obstetrics (3). The farmers were asked whether they noticed *retentio secundinarum* and how long the cows remained on straw after calving. 

### 2.5. Definitions

An NIMI was defined as the occurrence of a microorganism or a couple of microorganisms in a quarter milk sample after calving that were not present in the quarter milk sample on the day of drying off. If a pair of microorganisms was detected, a mixed intramammary infection was present. 

We defined a persistent intramammary infection in this study as the detection of the same genus or species of a microorganism in the milk sample before drying off and in the milk sample after calving.

### 2.6. Statistical Analysis

The collection and processing of the data were carried out with Microsoft Excel 2021 (Microsoft Corp., Redmond, WA, USA). To analyse the dataset, the program SPSS 28.0 (IBM, Inc., Chicago, IL, USA) was used. Risk factors for NIMI during the DP+ calving week were first analysed using univariable analysis. Univariable significant risk factors (*p* < 0.05) were then analysed in a generalised linear mixed model with logit link and binominal response (logistic regression). 

Two generalised linear mixed models were calculated. For the first generalised linear mixed model, each udder quarter counted as the statistical unit. For the second linear mixed model, only the rear udder quarters counted as the statistical unit. Backward stepwise procedures were used to select the final multivariable regression models. Potential risk factors were excluded if *p* > 0.05. Meaningful biological interactions between the fixed effects were also used in the final model if significant (*p* < 0.05) and if they did not increase the Akaike information criterion (AIC). Non-significant effects or interactions that increased the AIC were not included in the final models. Model fit was evaluated by checking the normality of the residuals. Odds ratios (OR) were calculated to compare risk factor groups with the reference group. The 95% confidence interval was calculated as well. Herd, cow and quarter were selected as random effects. A total of 12 quarters had no values for at least one significant risk factor and were therefore not included in the model. 

## 3. Results

### 3.1. Number of Cows and Quarters

A total of 848 quarters from 212 Holstein Friesian cows were examined for this study. Six cows died during the DP or shortly after calving, three cows were treated with antibiotics and two cows were no longer pregnant at the end of the DP. Therefore, 201 cows were included in the calculation. In addition, six quarters were excluded from this study because at least one quarter milk sample was contaminated; 798 quarters were conclusively considered.

### 3.2. Descriptive Results

The median age of the participating cows was 4.03 years (min. 2.08 to max. 11.01 years). The previous lactation lasted on average 332.5 days (min. 195 to max. 621 days). The median milk yield of the cows recorded in this study was 25.2 kg on the day before drying off. The lowest milk yield was 3.2 kg and the highest yielding cow produced 44.2 kg of milk on the day before drying off. A total of 19.3% of the examined cows had a milk yield below 15 kg, 16.0% milked 15–20 kg, and with 64.6% the majority exceeded 20 kg milk yield on the day before drying off. The duration of the DP was a median of 51 days, ranging from 12 days to 172 days. The number of quarters that showed no NIMI after calving and the number of quarters that showed an NIMI are listed in Table 3 and Table 4 depending on the risk factors investigated. 

### 3.3. Pathogens Isolated in Quarter Milk Samples before Drying Off, 7 ± 3 Days after Calving, and Pathogens Leading to NIMI during the DP+ Calving Week

On the day of drying off, quarter milk samples were taken from each cow participating in this study. The number of quarters with culture results before drying off and after calving are listed in Table 5. A total of 61.6% quarter milk samples showed no growth. In total, 314 quarter milk samples were found from which pathogens could be isolated. The most frequently isolated pathogens were coryneform bacteria (17.8% (151/848)), followed by NaS (13.1% (111/848)) and streptococci (1.8% (15/848)). A mixed infection was present in 3.3% (28/848) of the quarter milk samples. Gram-negative pathogens (coliform bacteria, *Serratia marcescens*) and other pathogens (*Trueperella pyogenes*, *Lactococcus gravieae*) were detected in 0.4% (3/848) and 0.5% (4/848) of the samples. Furthermore, *Staphylococcus aureus* was cultivated in 0.2% (2/848) of the quarter milk samples. A total of 7.1% (57/798) NIMI were detected by comparing the results of the quarter milk sample before drying off with the quarter milk sample 7 ± 3 days after calving. A total of 52.6% of the NIMI were detected in quarters that were microbiologically negative before drying off, and 47.4% of the NIMI were detected in already infected quarters at drying off with a different pathogen. The majority of NIMI were caused by NaS (40.4% (23/57)), followed by Gram-negative pathogens (22.8% (13/57)). Gram-negative pathogens were mainly *Pseudomonas* spp. (14.0% (8/57)), coliform bacteria (5.3% (3/57)) and *Acinetobacter* spp. (3.5% (2/57)). Streptococci caused 12.3% (7/57) of the NIMI, including *Streptococcus uberis* (7% (4/57)) and *Streptococcus dysgalactiae* (5.3% (3/57)). A total of 10.5% (6/57) of the NIMI were mixed infections, with two different pathogens. Other pathogens such as *Trueperella pyogenes* (3.5%), *Lactococcus garviae* (1.8%) and *Aerococcus viridans* (1.8%) caused 7.0% (4/57) of the NIMI. Coryneform bacteria and yeasts caused respectively 3.5% (2/57) of the NIMI during the DP+ calving week.

### 3.4. Udder Pressure

The udder pressure was measured five times. Mean highest udder pressure (0.663 kg/cm^2^) was reached 24 h after drying off. Thereafter, the udder pressure decreased; 72 h after drying off, the mean udder pressure (0.475 kg/cm^2^) still exceeded the base value (0.342 kg/cm^2^) after the last milking (Table 6). A significant correlation between udder pressure and milk yield on the day before drying off could be demonstrated. The highest correlation between the two variables was 24 h after drying off (R = 0.6); even 48 h and 72 h after drying off, there was a significant correlation (R = 0.5; R = 0.3).

### 3.5. Milk Leakage after Drying Off

During the 72 h of observation, 16.7% of the udder quarters showed milk leakage at least once (Table 7). The peak of milk leakage was observed 24 h after drying off (9.1%), and the majority of quarters started milk leakage 24 h after drying off (Table 7). However, 3.8% of the udder quarters showed milk leakage at more than one observation time (Table 8). By dividing the milk leakage into three grades, it was possible to observe that most of the udder quarters showed milk dripping from the teat end; milk flowing from the teat end was observed the least. 

Of the 142 udder quarters observed with milk leakage at least once, 14 quarters showed an NIMI after calving. NIMIs of the quarters with milk leakage were caused by NaS (50.0%), streptococci (21.4%), Gram-negative (14.3%), mixed infections (7.1%) and yeasts (7.1%). Quarters from cows with milk leakage had a significantly higher milk yield on the day before drying off (*p* < 0.0001). Cows with quarters leaking milk had an average milk yield of 28.32 kg on the day before drying off. In comparison, cows without quarters leaking milk produced 23.36 kg of milk on the day before drying off.

### 3.6. Udder Hygiene

Udders scored as free of dirt, score 1 and slightly dirty, score 2 are summarised as “clean udders”. Udders scored as covered with dirt, score 3 and covered with caked-on dirt, score 4 are referred to as “dirty udders” (Table 9).

NIMIs in quarters of cows with dirty udders were caused by NaS (50.0%), Gram-negative pathogens (20.0%), mixed intramammary infections (10.0%), Coryneform bacteria (10.0%) and other pathogens (10.0%).

### 3.7. Generalised Linear Mixed Model

A backward stepwise procedure was used to identify statistically significant risk factors for NIMI during the DP+ calving week. All univariable significant risk factors were eliminated one after another if *p* > 0.05. The results of the first generalised linear mixed model are presented in Table 10. The two remaining significant variables in the first generalised linear mixed model were udder hygiene 72 h after drying off (*p* < 0.001) and milk leakage 72 h after drying off (*p* = 0.012). 

Quarters that were observed with milk leakage 72 h after drying off had 3.4 higher odds (Cl 1.190–9.474) for NIMIs during the DP+ calving week compared to quarters without observed milk leakage. Quarters from cows with moderately dirt-covered udders (score 3) and caked-on dirt-covered udders (score 4) had 3.1 higher odds (CI 1.344–6.954) for NIMIs during the DP+ calving week compared to quarters from cows with udder hygiene score 1 and 2. Udders from those cows were free of dirt or slightly dirty. 

Results of the second generalised linear mixed model for rear quarters only are presented in Table 11. A second model was calculated because the udder pressure was just recorded for the rear quarters. A total of 404 quarters were included in this model. Statistically significant risk factors for NIMI in a rear quarter during the DP+ calving week were milk leakage 72 h after drying off (*p* = 0.008) and the duration of the DP (*p* = 0.010). Rear quarters showing milk leakage after 72 h had 5.8 higher odds (CI 1.576–21.396) for developing an NIMI during the DP+ calving week. 

## 4. Discussion

This study aimed to determine risk factors for NIMI during the DP+ calving week, with special attention to risk factors in the vulnerable phase of active involution and early lactation. Knowledge about risk factors for NIMI throughout the DP is mandatory to avoid CM at the beginning of the following lactation, thus preventing economic loss and starting the new lactation with a healthy mammary gland [5,7]. During the steady state, the DP offers the chance for the udder tissue to regenerate from the previous lactation, but initially, the udder tissue has to involute. The active involution is characterised by the transition from a lactating into a non-lactating udder tissue and starts with the accumulation of milk in the udder due to the omitted milking [14]. 

### 4.1. Udder Pressure

The highest udder pressure in the present study was measured 24 h after drying off. This finding is consistent with the readings of Bach et al. [17]. Contrary to these findings, Bertulat et al. [16] and Tucker et al. [52] detected the highest udder pressure on day two after drying off. A possible explanation for this deviation is that the extramammary udder pressure is determined by a variety of factors. The accumulating milk in the alveoli defines the intramammary udder pressure [53]. In addition to this, the extramammary udder pressure depends on the firmness of the udder tissue [48]. In addition, Tucker et al. [52] pointed out that cows milked once a day and cows that are fed less before drying off have lower udder pressure after drying off. A reduction in milking frequency and feed restriction are common methods of reducing milk yields. This supports our result that the udder pressure is associated with the milk yield before drying off. Bertulat et al. [54] showed that cows dried off abruptly showed the udder pressure peak on day one after drying off and those animals treated with Cabergoline to reduce milk production showed a peak in udder pressure on day two. This suggests that cows with a higher milk yield have a faster increase and thus an earlier peak in udder pressure. The study by Tucker et al. [52] included cows with a milk yield of 9.6 ± 2.9 kg per day, which is lower compared to the current study (25.2 kg). The different milk yields prior to drying off may explain the deviation in the udder pressure peak. The course of the udder pressure was uniform in these studies; the udder pressure peaked one or two days after drying off and then slowly decreased. The decreasing udder pressure is influenced by two factors: on the one hand, the high udder pressure leads to a loss of integrity of the mammary epithelium and thus stops further milk secretion; on the other hand, the liquid produced is reabsorbed after four days [15]. As presented, the pressure of an empty udder, such as after milking (base value), is not reached 72 h after drying off and the udder pressure can exceed the base value more than 9 days after drying off [16]. The udder pressure was no significant risk factor for NIMI during the DP+ calving week. Actually, high udder pressure is beneficial for the suppression of secretion. However, other factors are also being discussed that initiate the remodelling of the mammary tissue. In a study with goats, in addition to the high intramammary pressure, chemical factors in the milk that inhibit milk secretion and a hormone deficiency due to the lack of milking stimulus are discussed as reasons for the decline in milk secretion [15].

### 4.2. Milk Leakage after Drying Off and NIMI during the DP+ Calving Week

The high milk yield on the day of drying off makes abrupt drying off difficult nowadays and obviously leads to a rapidly increasing and higher udder pressure [16]. Moreover, every additional 5 kg of milk over 12.5 kg milk yield on the day of drying off increases the risk for NIMI at calving by 77% [32]. Although the milk yield was not statistically significant in the present study for NIMI during the DP+ calving week, it should be emphasised that the mean milk yield of all cows participating in the study was 25.2 kg on the day before drying off which clearly exceeds the 12.5 kg milk yield recommended by Rajala-Schultz et al. [32]. Only 19.3% of the examined cows had a milk yield below 15 kg and 64.6% of the cows exceeded 20 kg milk yield on the day before drying off. Nevertheless, we found that quarters with milk leakage belonged to cows with significantly more milk production before drying off (28.32 kg). Disadvantageous for a high milk yield before drying off is the lack of closure of the teat canal after drying off [19]. Physiologically, the keratin plug is formed as a physical barrier in the teat canal. Cows yielding over 21 kg before drying off had, according to Dingwell et al. [19], fewer closed teats during the DP. The teat canal is the interface between the environment and the udder tissue. If the teat canal is not closed properly, pathogens can penetrate the udder tissue. To protect healthy udder quarters from NIMI during the DP and support the teat canal closure, teat sealer was applied to every quarter in the present study. Nevertheless, 16.7% of the quarters were observed with milk leakage at least once, and milk leakage does indicate an insufficiently closed teat canal. We detected the milk leakage peak 24 h after drying off. Notably, udder pressure and milk leakage peaked 24 h after drying off, and that underlines the assumption of Rovai et al. [55] who detected a connection between large amounts of cisternal milk, which creates pressure and milk leakage. This study was carried out during lactation and was not related to the DP. However, the simultaneous peak of udder pressure and milk leakage may indicate a connection. In a study with dry cows, Bertulat et al. [16] also proved that a high udder pressure increases the risk of milk leakage (OR 3.35). Milk leakage prevalence peaked 24 h after drying off; 9.1% of the observed quarters showed milk leakage. In agreement with this, Bertulat et al. [54] described the highest milk leakage prevalence one day after drying off. Apart from that, De Prado-Taranilla et al. [20] detected the highest milk leakage incidence 36 h after drying off. They investigated those cows for milk leakage 20–24 h, 30–34 h and 48–52 h after drying off. The timeframe 30–34 h after drying off was not considered in the present study and therefore it is possible that several quarters with milk leakage were missed. This means that the udder pressure could also peak at 36 h, assuming that udder pressure and milk leakage peak at the same time. Another difference between the two studies was the length of the observation. We additionally inspected the cows for milk leakage 72 h after drying off. Neither of the studies had a permanent monitoring of the cows, which might explain the difference in the milk leakage peak. De Prado-Taranilla et al. [20] observed 9.3% quarters leaking milk at least once during the DP, presenting fewer quarters with milk leakage than the present study (16.7%), even though 85.4% of the examined herds did not apply a teat sealer as a drying-off treatment. A further explanation for the differences is that milk leakage depends on several factors. Rovai et al. [55] investigated reasons for milk leakage during lactation. They observed that a high milk flow and short teats with short teat canals lead more frequently to milk leakage [55]. This thesis was also supported in a study by Klaas et al. [56] which showed that shorter teats with short teat canals are a risk factor for milk leakage. However, there are different results regarding the lactation number. Bach et al. [17] constitute that multiparous cows are more susceptible to milk leakage, whereas Rovai et al. [55] observed low-performing primiparous cows with a high milk flow with higher milk leakage incidence. The interaction of risk factors with the muscle tone of the teat sphincter is likely to be decisive for milk leakage [56]. Regardless of the milk leaking prevalence, quarters showing milk leakage had 3.4 higher odds for NIMI during the DP+ calving week. Quarters leaking milk 72 h after drying off were at greater risk for NIMI. That implies that quarters that are not only open after drying off, but also open over a longer time, have a greater risk for NIMI during the DP. It can be assumed that udder quarters which are open for a longer time are exposed for longer and that contact with pathogens is more likely. Our findings are in agreement with those of De Prado-Taranilla et al. [20] who proved that cows with milk leakage tended to have 1.5 higher odds of developing an NIMI during the DP or up to 30 d after calving.

In our study, 7.1% NIMI could be confirmed during the DP+ calving week. De Prado-Taranilla et al. [20] presented 22.3% NIMI in their study. According to that, fewer NIMIs occurred despite a detection of a higher milk leakage prevalence. However, it should be noted that De Prado-Taranilla et al. [20] defined an NIMI as CM during the DP and in the first 30 days of lactation or an increased individual somatic cell count (ISCC) after the DP compared to ISCC before drying off. In the present study, an NIMI was defined as the detection of a microorganism or a couple of microorganisms in a quarter milk sample 7±3 days after calving that was not cultivated in the quarter milk sample before drying off. The study by Nitz et al. [4] pointed out that an additional 5.1% NIMI occurred 17 ± 3 days after calving, independently from the DP. The deviating number of NIMIs can therefore be attributed to the different definitions of NIMI and also to the different sampling period. Based on the results of Nitz et al. [4], it is comprehensible that De Prado-Taranilla et al. [20] was able to detect more NIMIs due to the longer sampling period after calving. The second sampling date in this study was set 7 ± 3 days after calving and thus ranged from 4 days to 10 days after calving. A reliable mastitis diagnosis is possible from day 3 after calving [24]. To detect only the NIMIs that occurred during the DP, the sampling period should have taken place within a shorter sampling interval. However, as all samples were only taken by one veterinarian, the second sampling date had an interval of six days. During these six days, NIMIs could have developed independently of the DP. It should also be noted that teat canals can reopen early before parturition and are therefore, as open teat canals at the beginning of the DP, a risk factor for NIMI during the DP [57]. However, periparturient cows were not examined in this study.

Compared to other studies that also used long-lasting antibiotics as dry-off treatment and microbiological examinations to verify an NIMI, fewer NIMIs occurred in the present study. Arruda et al. [28] detected 13.3% and Gundelach et al. [1] 20.7% NIMIs during the DP or the first days after calving. Differences can be explained by the multifactorial development of NIMIs [21]. Two studies from Krömker et al. [3,58] which included only healthy udder quarters at drying off showed 7.4% and 3.4% of NIMI, respectively, during the DP or the first days after calving, with a teat sealer being the only drying-off treatment. The control groups in those studies that received no dry-off treatment showed 12.7% and 10.5% of NIMIs, respectively, during the DP or the first days after calving. Those two studies highlight the importance of udder health before drying off [3,58]. In the present study, all quarters were included regardless of udder health before drying off. Nonetheless, the low rate of NIMI indicates good udder health on the participating farms (7.1%).

Most NIMIs during the DP+ calving week were caused by NaS (40.4%). That is consistent with the findings of other studies like Nitz et al. [4], Krömker et al. [58] and Piepers et al. [59]. Piepers et al. [59] suspected a protective effect through the colonisation of the teat apex with CNS (coagulase-negative staphylococci), which leads to the prevention of infection with major mastitis pathogens. If coryneform bacteria are also considered, 43.9% of the NIMIs were caused by minor pathogens. Cow-associated mastitis pathogens (*Streptococcus dysgalactiae*, *Streptococcus agalactiae*, *Staphylococcus aureus*) led to 5.3% of NIMIs. 

Environmental pathogens like *Pseudomonas* spp. (14.0%), *Streptococcus uberis* (7.0%) and coliform bacteria (5.3%) also caused NIMIs. Nitz et al. [4] demonstrated a connection between teat end hyperkeratosis and NIMI with environmental pathogens, suspecting an insufficient closure of the teat canal due to the ring of hyperkeratosis. Teat end hyperkeratosis was not statistically significant in the present study for NIMI. 

### 4.3. Udder Hygiene and NIMI during the DP+ Calving Week

In line with the results of this study, NIMIs during the DP with environmental pathogens are more likely to occur than those with contagious pathogens. Contagious pathogens are transferred from one infected cow to other cows during milking. Environmental pathogens are mainly found in the cow’s barn [60]. Especially, coliform bacteria and environmental streptococci are transmitted via the manure and bedding material [44]. To compare the cleanliness of the cows in our study, the udder hygiene was assessed five times. Dirt is either transferred directly to the udder via splashes or through transmission from the legs and tail [44]. Dry cows were separated from the herd and no longer had to walk to the milking parlour several times a day. The deviating daily routine, management and environment of dry cows might explain that more udders were scored as “clean udders” after drying off compared to before drying off and after calving. Furthermore, the different feed rations change the consistency of the dry cow’s faeces, and the cleanliness of the udders is significantly related to the faecal consistency [61]. Even though more udders were scored as “clean udders” after drying off, cows with udder hygiene scores 3 and 4 had 3.1 higher odds for an NIMI in contrast to cows with clean or slightly dirty udders 72 h after drying off. In agreement with our findings, Schreiner and Ruegg [35] found a correlation between the occurrence of contagious and environmental pathogens in the udder and the udder hygiene in lactating cows. They detected major pathogens 1.5 times more frequently in cows with an udder hygiene score of 3 and 4. Furthermore, Guarín et al. [62] found higher bacterial counts on teat skins of cows with udder hygiene scores 3 and 4. 

### 4.4. Duration of the Dry Period

Several studies have looked at the effects of different lengths of dry periods. Church et al. [31] compared three groups of cows with 30-, 45-, and 60-day long dry periods. They found no negative effect on udder health due to the different DP lengths. This can be explained by the fact that a longer DP prolongs the phase of the steady state, whereas active involution and neo-lactogenesis, which are the hazardous periods concerning udder health, remain the same length of time and are not influenced by the duration of the DP [14]. In contrast, our results indicate that a longer DP (>45 days) increases the risk of an NIMI. These results agree with those of Dingwell et al. [30] who justified their results by stating that a prolonged DP also extended the time during which a quarter of the udder is exposed to pathogens. In addition, the concentration of the active constituents of the long-acting antibiotic decreases towards the end of the DP, falling below the minimum inhibitor concentration, and no longer provides any protection at the end of long dry periods [63]. The concentration of active constituents would only be sufficient in very short dry periods, but a short DP would have negative effects on the milk yield of the subsequent lactation [31]. Nevertheless, the cows in the trial had a higher risk of NIMI in the rear quarters if the DP was longer than 45 days. However, the broad confidence intervals in the second generalised linear mixed model suggest a more uncertain effect size estimate. 

### 4.5. Limitations of This Study

The cross-sectional study has been carried out between August and January. The cows were therefore dried off but also calved under very different climatic conditions. Heat stress can influence the immune system and therefore more NIMIs may have developed in the summer [64]. To detect an NIMI of an udder quarter, duplicate quarter milk samples were taken and the results of the samples after calving were compared with those before drying off. The detection of a different microorganism or a couple of microorganisms in the second quarter milk sample thus confirmed an NIMI. To reliably detect an NIMI, an additional quarter milk sample after calving at a one-week interval would have been beneficial to isolate the pathogen at least twice [65]. The number of NIMIs was therefore perhaps overestimated. Compared with similar studies, however, the number of NIMIs detected in the current study was already low [1,28]. 

As mentioned above, milk leakage was observed 24 h, 48 h and 72 h after drying off. Permanent monitoring of the udder quarters would be necessary to ensure that no quarter with milk leakage is overlooked. This study was therefore only able to make a statement concerning the actual observed times and the prevalence of milk leakage may have been underestimated. In addition, udder quarters could be observed for milk leakage at various times to gain a more precise impression of milk leakage. Moreover, udder quarters should be observed for milk leakage during the periparturient period, as the udder tissue is also susceptible to NIMI at this time [4,14]. It should also be noted that all cows in the trial were treated with a long-acting intramammary antibiotic for drying off. Antibiotic treatment as a dry-off treatment is particularly important for the cure of existing intramammary infections. And additionally, the aim of our study was to investigate risk factors for the development of NIMI independently of the dry-off treatment. The effects of the dry period length on udder health should be investigated in more detail in further studies. 

## 5. Conclusions

The presented study ascertained risk factors for NIMI during the DP+ calving week. Quarters from cows with dirty udders (scores 3 and 4) after drying off were more at risk of developing an NIMI during the DP+ calving week than quarters from cows with clean udders (scores 1 and 2). This underlines the importance of hygiene in the cow’s environment to minimise contact with pathogens, especially because the daily milking and teat disinfection do not take place during the DP. 

Additionally, teats with observed milk leakage had 3.4 times higher odds of developing an NIMI during the DP+ calving week compared to teats without observed milk leakage. The high milk leakage prevalence (16.7%) illustrates the inadequate closure of the teat canal after drying off. Since we found a correlation between the milk yield before drying off and milk leakage, milk leakage should be prevented by a reduction in milk yield before drying off. 

Despite the high observed milk leakage prevalence of 16.7%, the verified 7.1% NIMI occurring during the DP+ calving week were comparatively low. 

This study emphasises the need to adapt dry cow management when high milk yields occur and the importance of hygiene after drying off to avoid NIMI during the DP. Thus, the risk factors before drying off and shortly after drying off were decisive for the development of NIMI, whereas the investigated risk factors during the periparturient period were not decisive for the development of NIMI on the participating farms.

## Figures and Tables

**Table 1:** Literature research on cow-associated risk factors for the development of NIMI ^1^.

Risk Factor	Reference	Extended Dry Period ^2^	Results
Lactation number	Arruda et al., 2013 [28]	Yes	Increasing parity (>2) associated with the risk of developing an NIMI ^1^ (OR ^3^ 0.78)
	Gundelach et al., 2011 [1]	Yes	Udder quarters from cows with >4 lactations higher risk for NIMI ^1^
Duration of lactation	Robert et al., 2008 [29]	Yes	Longer previous lactation (>355 days) associated with a higher risk for NIMI ^1^ vs. shorter lactation length (305–355 days)
Duration of DP ^4^	Robert et al., 2008 [29]	Yes	Longer DP ^4^ (>65 days) associated with risk for NIMI ^1^ vs. shorter DP ^4^ (<65 days)
	Dingwell et al., 2002 [30]	Yes	Increased risk for NIMI ^1^ when the DP ^4^ was longer (OR ^3^ 1.1)
	Church et al., 2008 [31]	Yes	No significant differences between duration of DP ^4^ (30, 45, 60 days) and development of NIMI ^1^
Milk yield	Rajala-Schultz et al., 2005 [32]	Yes	Milk yield at dry-off increased risk for NIMI ^1^ with environmental pathogens at calving every 5 kg above 12.5 kg of milk
	Dingwell et al., 2002 [30]	Yes	Higher milk yield before drying off increased risk for NIMI (OR ^3^ 1.3)
305-day milk production	Green et al., 2007 [33]	Yes	305-day milk yield no risk factor for CM ^5^ after calving
Milking frequency	Gott et al., 2016 [34]	Yes	Multiparous cows milked three times daily during lactation increased odds of NIMI ^1^ occurring at calving compared to cows milked twice daily (OR ^3^ 3.4)
Udder hygiene	Schreiner and Ruegg, 2003 [35]	No	Major pathogens more frequently detected in milk samples of cows with dirty udders (scores 3 and 4 ^6^) than in milk samples from clean udders (scores 1 and 2 ^6^) (OR ^3^ 1.5)
	Compton et al., 2007 [22]	Yes	Udder hygiene scores ≥ 2 associated with NIMI ^1^ with environmental pathogens after calving compared to udder hygiene Score 1 ^6^
Udder oedema after calving	Nitz et al., 2020 [36]	Yes	Heifers with persistent strong udder oedema increased risk for NIMI ^1^ with NaS ^7^ and coryneform bacteria vs. heifers without udder oedema (OR ^3^ 4.3)
	Krömker et al., 2012 [23]	No	Present udder oedema was a significant risk factor for NIMI ^1^ (OR ^3^ 1.78)
BCS ^8^	Nitz et al., 2021 [4]	Yes	Consistent BCS ^8^ decreased risk for NIMI ^1^ (OR ^3^ 0.39) BCS ^8^ <3.5 decreased risk for NIMI ^1^ with NaS ^7^ and coryneform bacteria (OR ^3^ 0.45)
	Gundelach et al., 2011 [1]	Yes	BCS ^8^ <3.0 and >3.5 higher risk for CM ^5^
Lameness	Singh et al., 2018 [37]	No	Lame cows showed a higher milk SCC ^9^ than non-lame cows
*Retentio secundinarium*	Schukken et al., 1988 [38]	No	Cows with retained placenta had a higher risk of developing severe mastitis after calving (OR ^3^ 5.4)
Days on straw after calving	Peeler et al., 2000 [39]	No	Incidence for CM ^5^ increased when cows housed in straw yards compared to cubicle housing
	Green et al., 2007 [33]	Yes	Hygiene measures in calving areas are significantly associated with increased risk for CM ^5^

^1^: new intramammary infection; ^2^: whether the cited study was conducted during the extended dry period; ^3^: odds ratio; ^4^: dry period; ^5^: clinical mastitis; ^6^: udder hygiene score by Schreiner and Ruegg, 2002 [40]; ^7^: non-*aureus* Staphylococci; ^8^: body condition score; ^9^: somatic cell count.

**Table 2:** Literature research on quarter-associated risk factors for the development of NIMI ^1^.

Risk factor	Reference	Extended Dry Period ^2^	Results
Present IMI ^3^ at drying off	Robert et al., 2008 [29]	Yes	Cows with IMI ^3^ in one quarter at drying off significantly associated with the risk for NIMI ^1^ compared to uninfected cows at drying off
	Gott et al., 2016 [34]	Yes	Presence of IMI ^3^ on the day of drying off increased risk for NIMI ^1^ at calving
	Newman et al., 2010 [41]	Yes	Infected quarters at drying off had higher odds of NIMI ^1^ occurring with major pathogens (OR ^4^ 7.6) and minor pathogens (OR ^4^ 3.3) compared to uninfected quarters at drying off
Milk leakage (ML ^5^)	De Prado-Taranilla et al., 2020 [20]	Yes	Cows with ML ^5^ tended to have higher odds of NIMI ^1^ occurring (OR ^4^ 1.5)
	Gott et al., 2016 [34]	Yes	Quarters of primiparous cows with ML had higher odds of NIMI ^1^ occurring at calving compared to quarters without milk leakage (OR ^4^ 28.9)
Teat cleanliness	Roberson et al., 1994 [42]	Yes	Heifers with teat skin colonisation with *S. aureus* more likely to develop NIMI ^1^ with *S. aureus* after calving compared to non-colonised quarters (OR ^4^ 3.3)
Teat end condition	Nitz et al., 2021 [4]	Yes	Absence of hyperkeratosis at teat apex lowered risk for NIMI ^1^ vs. hyperkeratosis present at teat apex (OR ^4^ 0.38)
	Dingwell et al., 2004 [19]	Yes	Rough or cracked teat ends had higher odds of NIMI ^1^ occurring than teats without cracks (OR ^4^ 2.5)
Teat morphologies	Krömker et al., 2012 [23]	No	Teat length < 35 mm; diameter < 18 mm associated with NIMI ^1^ in heifers during first 41 DIM ^6^ (OR ^4^ 2.81; OR ^4^ 2.45)
	Compton et al., 2007 [22]	Yes	Low minimum teat height (<53 cm) above the ground risk factor for subclinical mastitis after calving
Teat end shape	Abebe et al., 2016 [43]	No	Flat or round teat ends more likely to have mastitis vs. pointed teat ends (OR ^4^ 7.6; OR ^4^ 3.2)

^1^: new intramammary infection; ^2^: whether the cited study was conducted during the extended dry period; ^3^: intramammary infection; ^4^: odds ratio; ^5^: milk leakage; ^6^: days in milk.

**Table 3:** Cow-level associated independent variables for NIMI ^1^ during the DP+ calving week ^2^.

Independent Variable	N of Quarters without NIMI ^1^ (%): 741 Quarters	N of Quarters with NIMI ^1^ (%): 57 Quarters
Lactation number		
1	232 (31.3)	16 (28.1)
2	241 (32.5)	10 (17.5)
≥3	268 (36.2)	31 (54.4)
Milking frequency		
2	255 (34.4)	26 (45.6)
3	486 (65.6)	31 (54.4)
Milk yield		
<15 kg	141 (19.0)	10 (17.5)
15–20 kg	115 (15.5)	8 (14.0)
>20 kg	485 (65.5)	39 (68.4)
Lameness		
normal	570 (76.9)	39 (68.4)
mildly lame	118 (15.9)	12 (21.1)
moderately to severely lame	53 (7.2)	6 (10.5)
BCS ^3^		
<3.5	339 (45.7)	28 (49.1)
≥3.5	402 (54.3)	29 (50.9)
Difference in BCS ^4^		
no difference	200 (27.0)	16 (28.1)
negative	386 (52.1)	32 (56.1)
positive	147 (19.8)	9 (15.8)
missing	8 (1.1)	
*Retentio secundinarium*		
no	673 (90.8)	46 (80.7)
yes	68 (9.2)	11 (19.3)
Udder oedema		
no	441 (59.5)	33 (57.9)
yes	297 (40.1)	23 (40.4)
missing	3 (0.4)	1 (1.8)
Days on straw after calving		
0 days	460 (62.1)	29 (50.9)
1–5 days	132 (17.8)	11 (19.3)
>5 days	114 (15.4)	12 (21.1)
missing	35 (4.7)	5 (8.8)

^1^: new intramammary infection; ^2^: extended dry period between drying off and 7 ± 3 days after calving; ^3^: body condition score; ^4^: comparison of the BCS on the day of drying off and on the second sampling date after calving, whereas a negative change describes a decline in BCS by at least 0.25 increments and a positive change describes an increase in BCS by at least 0.25 increments; the figures do not add up to 100 due to rounding.

**Table 4:** Quarter-level associated independent variables for NIMI ^1^ during the DP+ calving week ^2^.

Independent Variable	N of Quarters without NIMI ^1^ (%): 741 Quarters	N of Quarters with NIMI ^1^ (%): 57 Quarters
Teat cleanliness		
1	141 (19.0)	10 (17.5)
2	561 (75.7)	46 (80.7)
missing	39 (5.3)	1 (1.8)
Teat end condition		
no ring, smooth ring	388 (52.4)	23 (40.4)
rough ring, very rough ring	353 (47.6)	34 (59.6)

^1^: new intramammary infection, ^2^: extended dry period between drying off and 7 ± 3 days after calving; the figures do not add up to 100 due to rounding.

**Table 5:** Number of quarters with culture results of the quarter milk samples on the day of drying off, 7 ± 3 days after calving, and number of quarters with a NIMI ^1^ developed during the DP+ calving week ^2^; and the respective culture results.

	Number (%) of Quarters with Culture Results		
Culture Result	On the Day of Drying Off	7 ± 3 Days after Calving	NIMI ^1^
NaS ^3^	111 (13.1)	23 (2.9)	23 (40.4)
*Corynebacterium* spp.	151 (17.8)	4 (0.5)	2 (3.5)
Gram-negative pathogens total	3 (0.4)	14 (1.7)	13 (22.8)
Coliform bacteria ^4^	2	3	3
*Serratia marcescens*	1		
*Pseudomonas* spp.		9	8
*Acinetobacter* spp.		2	2
Streptococci total	15 (1.8)	7 (0.9)	7 (12.3)
*Enterococcus* spp.	3		
*Streptococcus dysgalactiae*	4	3	3
*Streptococcus uberis*	7	4	4
Other streptococci	1		
Mixed infections	28 (3.3)	9 (1.1)	6 (10.5)
Other pathogens ^5^	4 (0.5)	4 (0.5)	4 (7.0)
*Staphylococcus aureus*	2 (0.2)		
Yeasts		2 (0.2)	2 (3.5)
Contaminated	12 (1.4)	2 (0.2)	
No growth	522 (61.6)	739 (91.9)	
In total ^6^	848 (100.0)	804 (100.0)	57 (100.0)

^1^: new intramammary infection; ^2^: extended dry period between drying off and 7 ± 3 days after calving; ^3^: non-*aureus* staphylococci; ^4^: coliform bacteria: *Escherichia coli, Klebsiella pneumoniae, Enterobacter* spp.; ^5^: other pathogens: *Trueperella pyogenes*, *Lactococcus gravieae, Aerococcus viridans*; ^6^: total number of quarters 7 ± 3 days after calving is lower than on the day of drying off, because 44 quarters could not be sampled on the second sampling date; the figures do not add up to 100 due to rounding.

**Table 6:** Udder pressure development before and after drying off.

	Mean	Minimum	Maximum	N ^6^	SD ^7^
ED1 ^1^	0.604 kg/cm^2^;	0.2 kg/cm^2^;	1.5 kg/cm^2^;	404	0.2115
ED2 ^2^	0.342 kg/cm^2^;	0.2 kg/cm^2^;	0.8 kg/cm^2^;	424	0.1224
ED24 ^3^	0.663 kg/cm^2^;	0.2 kg/cm^2^;	1.5 kg/cm^2^;	424	0.2307
ED48 ^4^	0.606 kg/cm^2^;	0.2 kg/cm^2^;	1.6 kg/cm^2^;	424	0.2169
ED72 ^5^	0.475 kg/cm^2^;	0.2 kg/cm^2^;	1.0 kg/cm^2^;	408	0.1779

^1^: udder pressure before the last milking; ^2^: udder pressure after the last milking (base value); ^3^: udder pressure 24 h after drying off; ^4^: udder pressure 48 h after drying off; ^5^: udder pressure 72 h after drying off; ^6^: number of quarters; ^7^: standard deviation.

**Table 7:** The time points of quarters showing milk leakage for the first time after drying off.

	Number (%) of Quarters		
	24 h after Drying Off	48 h after Drying Off	72 h after Drying Off
Milk leakage			
yes	77 (9.1)	50 (5.9)	15 (1.8)
no	771 (90.9)	798 (94.1)	833 (98.2)
In total	848 (100.0)	848 (100.0)	848 (100.0)

**Table 8:** Number of quarters showing milk leakage at each observed time point.

	Number (%) of Quarters		
	24 h after Drying Off	48 h after Drying Off	72 h after Drying Off
Milk leakage			
yes	77 (9.1)	70 (8.3)	27 (3.2)
no	771 (90.9)	778 (91.7)	821 (96.8)
In total	848 (100.0)	848 (100.0)	848 (100.0)

**Table 9:** Udder hygiene scores before drying off, during the early dry period and after calving.

	Number (%) of Udders				
	Day of Drying Off	24 h after Drying Off	48 h after Drying Off	72 h after Drying Off	7 ± 3 Days Postpartum
Udder hygiene					
clean udders ^1^	136 (64.2)	193 (91.5)	192 (91.0)	190 (92.7)	135 (67.5)
dirty udders ^2^	76 (35.8)	18 (8.5)	19 (9.0)	15 (7.3)	65 (32.5)
In total	212 (100.0)	211 (100.0)	211 (100.0)	205 (100.0)	200 (100.0)

^1^: udder hygiene scores 1 and 2; ^2^: udder hygiene scores 3 and 4; the figures do not add up to 100 due to rounding.

**Table 10:** Results of the first generalised linear mixed model for the probability of a quarter to develop an NIMI ^1^ during the DP+ calving week ^2^.

Dependent Variable	Independent Variable (Reference Category)	ß ^3^	SE ^4^	OR ^5^	95%CI ^6^ (OR)	*p*-Value
NIMI ^1^ between drying off and 7 ± 3 days postpartum	Quarters observed with ML ^7^ 72 h after drying off (quarters without observed ML)	1.211	0.5285	3.357	1.190–9.474	0.022
NIMI ^1^ between drying off and 7 ± 3 days postpartum	Quarters from udders with hygiene scores 3 and 4, 72 h after drying off (hygiene scores 1 and 2)	1.117	0.4187	3.057	1.344–6.964	0.008

^1^: new intramammary infection; ^2^: extended dry period between drying off and 7 ± 3 days after calving; ^3^: regression coefficient; ^4^: standard error of the mean; ^5^: odds ratio; ^6^: 95% confidence interval; ^7^: milk leakage.

**Table 11:** Results of the second generalised linear mixed model for the probability of a rear quarter to develop an NIMI ^1^ during the DP+ calving week ^2^.

Dependent Variable	Independent Variable (Reference Category)	ß ^3^	SE ^4^	OR ^5^	95%CI ^6^ (OR)	*p*-Value
NIMI ^1^ between drying off and 7 ± 3 days postpartum	Quarters observed with ML ^7^ 72 h after drying off (quarters without observed ML ^7^)	1.759	0.6632	5.807	1.576–21.396	0.008
NIMI ^1^ between drying off and 7 ± 3 days postpartum	Quarters from cows with DP ^2^ length 40–49 days (quarters from cows with DP ^2^ length <40 days)	2.530	3.0892	12.555	0.29–5455.732	0.413
	Quarters from cows with DP ^2^ length 50–59 days (quarters from cows with DP ^2^ length <40 days)	4.066	3.0720	58.317	0.139–24,501.511	0.186
	Quarters from cows with DP ^2^ length 60–69 days (quarters from cows with DP ^2^ length <40 days)	4.744	3.0856	114.837	0.266–49,554.061	0.125
	Quarters from cows with DP ^2^ length 70–79 days (quarters from cows with DP ^2^ length <40 days)	2.628	3.2373	13.851	0.24–8054.966	0.417
	Quarters from cows with DP ^2^ length ≥80 days (quarters from cows with DP ^2^ length <40 days)	3.563	3.1478	35.260	0.72–17,195.088	0.258

^1^: new intramammary infection; ^2^: extended dry period between drying off and 7 ± 3 days after calving; ^3^: regression coefficient; ^4^: standard error of the mean; ^5^: odds ratio; ^6^: 95% confidence interval; ^7^: milk leakage.

## Data Availability

Dataset available on request from the authors.
